# Application of Common Culinary Herbs for the Development of Bioactive Materials

**DOI:** 10.3390/plants13070997

**Published:** 2024-03-30

**Authors:** Alina Ioana Lupuliasa, Anda-Maria Baroi, Sorin Marius Avramescu, Bogdan Stefan Vasile, Răzvan Mihai Prisada, Radu Claudiu Fierascu, Irina Fierascu, Daniela Ionela Sărdărescu (Toma), Alexandra Ripszky Totan, Bianca Voicu-Bălășea, Silviu-Mirel Pițuru, Lăcrămioara Popa, Mihaela Violeta Ghica, Cristina-Elena Dinu-Pîrvu

**Affiliations:** 1 Department of Physical and Colloidal Chemistry, Faculty of Pharmacy, “Carol Davila” University of Medicine and Pharmacy, 6 Traian Vuia Str., 020956 Bucharest, Romania; alina-ioana.lupuliasa@drd.umfcd.ro (A.I.L.); lacramioara.popa@umfcd.ro (L.P.); mihaela.ghica@umfcd.ro (M.V.G.); cristina.dinu@umfcd.ro (C.-E.D.-P.); 2 National Institute for Research & Development in Chemistry and Petrochemistry ICECHIM Bucharest, 202 Splaiul Independenței, 060021 Bucharest, Romania; anda.baroi@icechim.ro (A.-M.B.); irina.fierascu@icechim.ro (I.F.); 3 Faculty of Horticulture, University of Agronomic Sciences and Veterinary Medicine of Bucharest, 59 Mărăști Blvd., 011464 Bucharest, Romania; 4 Department of Inorganic Chemistry, Organic Chemistry, Biochemistry and Catalysis, Faculty of Chemistry, University of Bucharest, 030018 Bucharest, Romania; sorin.avramescu@g.unibuc.ro; 5 Research Centre for Environmental Protection and Waste Management (PROTMED), University of Bucharest, Splaiul Independenței 91-95, Sect. 5, 050107 Bucharest, Romania; 6 Research Center for Advanced Materials, Products and Processes, National University of Science and Technology POLITEHNICA Bucharest, 313 Splaiul Independenţei, 060042 Bucharest, Romania; bogdan.vasile@upb.ro; 7 National Research Center for Micro and Nanomaterials, National University of Science and Technology POLITEHNICA Bucharest, 313 Splaiul Independenţei, 060042 Bucharest, Romania; 8 Faculty of Chemical Engineering and Biotechnology, National University of Science and Technology Politehnica Bucharest, 1-7 Gheorghe Polizu St., 011061 Bucharest, Romania; ionela.toma93@yahoo.com; 9 National Research and Development Institute for Biotechnology in Horticulture, 37 Bucureti-Pitesti Str., 117715 Ștefănești, Romania; 10 Department of Biochemistry, Faculty of Dental Medicine, “Carol Davila” University of Medicine and Pharmacy, 17-23 Plevnei Street, 020021 Bucharest, Romania; alexandra.ripszky@umfcd.ro; 11 Interdisciplinary Center for Dental Research and Development, “Carol Davila” University of Medicine and Pharmacy, 6 Traian Vuia Str., 020956 Bucharest, Romania; bianca.voicu-balasea@drd.umfcd.ro (B.V.-B.); silviu.pituru@umfcd.ro (S.-M.P.); 12 Department of Organization, Professional Legislation and Management of the Dental Office, Faculty of Dental Medicine, “Carol Davila” University of Medicine and Pharmacy, 17-23 Plevnei Street, 020021 Bucharest, Romania; 13 Innovative Therapeutic Structures R&D Center (InnoTher), “Carol Davila” University of Medicine and Pharmacy, 6 Traian Vuia Str., 020956 Bucharest, Romania

**Keywords:** hyssop, oregano, phytosynthesis, silver nanoparticles, antioxidant, cell viability, inflammation level, cytotoxicity

## Abstract

Hyssop (*Hyssopus officinalis* L.) and oregano (*Origanum vulgare* L.), traditionally used for their antimicrobial properties, can be considered viable candidates for nanotechnology applications, in particular for the phytosynthesis of metal nanoparticles. The present work aims to evaluate the potential application of hyssop and oregano for the phytosynthesis of silver nanoparticles, as well as to evaluate the biological activities of their extracts and obtained nanoparticles (antioxidant potential, as well as cell viability, inflammation level and cytotoxicity in human fibroblasts HFIB-G cell line studies). In order to obtain natural extracts, two extraction methods were applied (classical temperature extraction and microwave-assisted extraction), with the extraction method having a major influence on their composition, as demonstrated by both the total phenolic compounds (significantly higher for the microwave-assisted extraction; the oregano extracts had consistently higher TPC values, compared with the hyssop extracts) and in terms of individual components identified via HPLC. The obtained nanoparticles ware characterized via X-ray diffraction (XRD) and transmission electron microscopy (TEM), with the lowest dimension nanoparticles being recorded for the nanoparticles obtained using the oregano microwave extract (crystallite size 2.94 nm through XRD, average diameter 10 nm via TEM). The extract composition and particle size also influenced the antioxidant properties (over 60% DPPH inhibition being recorded for the NPs obtained using the oregano microwave extract). Cell viability was not affected at the lowest tested concentrations, which can be correlated with the nitric oxide level. Cell membrane integrity was not affected after exposure to classic temperature hyssop extract-NPs, while the other samples led to a significant LDH increase.

## 1. Introduction

Culinary spices, beyond their traditional role in enhancing the flavor and aroma of food, hold significant importance in modern science for various reasons. Many culinary spices have been recognized for their potential health benefits due to the presence of bioactive compounds. For example, turmeric contains curcumin, which has anti-inflammatory and antioxidant properties. These compounds are of interest in medical research for their potential in preventing and treating various diseases [1]. Among the various applications, the development of new nanotechnological instruments mediated by these herbs represents a promising area for future research [2]. 

Many of the herbal preparations used in traditional medicine are based on folklore, rather than scientific foundation, and this also applies to hyssop, underlining its biomedical potential [3].

The genus Hyssopus comprises herbaceous perennials or subshrubs that are mainly cultivated but can also be found in the wild. The genus comprises about 10–12 species, distributed mainly in the eastern Mediterranean and Central Asia. *H. officinalis* L. has a long history of medicinal use as a carminative, tonic, antiseptic, cough expectorant. However, although it has a slightly bitter taste, *H. officinalis* is often used as a flavor and spice in the food industry. The benefits of the traditional use of this plant are supported by several previous studies, which identified several bioactive compounds, especially the main compounds in the essential oils [4]. A phytochemical study conducted on China-native *H. officinalis* plants revealed the presence of two new flavonoid glycosides and nine other known flavonoids from the ethanolic extract of the plant, with radical-scavenging activity superior to butylated hydroxytoluene and L-ascorbic acid (used as standards). Also, the study showed that the methanolic extract of *H. officinalis* is rich in phenolic compounds, particularly in chlorogenic, protocatechuic, ferulic, syringic, p-hydroxybenzoic and caffeic acids, followed by vanillic, p-coumaric, rosmarinic and gentisic acids [5]. Other previous studies reported the presence of diosmin and other flavonoids in this plant [6,7].

Ahmadian et al. [8] evaluated the antioxidant potential of a *H. officinalis* extract obtained via ultrasound-assisted extraction (accompanied by a cold atmospheric plasma pre-treatment), observing a significant influence of the pre-treatment and extraction method on the total phenolic content, as well as on the antioxidant potential. Hydroalcoholic *H. officinalis* extracts were proven to have preventive effects in improving seizures, while also improving post-seizure memory deficits [9].

In another study, the essential oil and methanol extracts of *H. officinalis* were tested for their antioxidant and antimicrobial activities in vitro. The essential oil showed activity against eight bacteria and ten fungi, including *C. albicans*, but no distinct antioxidant properties were observed [10]. 

Ebrahimzadeh et al. [11] used six different *in vitro* methods to evaluate the antioxidant and free-radical-scavenging activities of the methanolic extract of the aerial parts of *H. officinalis*. They found that the methanolic extract exhibited modest-to-potent antioxidant activities in DPPH radical reduction and scavenging, as well as Fe^2+^ chelation and assay capacity [11]. 

The antifungal property of 12 essential oils from Mediterranean plants against four fungi was investigated, concluding that hyssop oil exhibits weak-to-moderate antifungal activity. Other studies examined the antifungal and insecticidal properties of the plant, observing the complete inhibition of the growth of the fungus *Aspergillus niger* at concentrations of 0.5 to 1.5% *v*/*v*, while the methanolic extract showed a certain degree of larval toxicity in the case of the Egyptian cotton bollworm (*Spodoptera littoralis*) [12,13,14]. Zinc oxide nanoparticles synthesized using *Hyssopus officinalis* L. extracts were presented by Rahimi et al. [15] to be toxic in mice models, at 50 mg/kg or higher concentrations.

The genus *Origanum* consists of an important variety of medicinal and aromatic plants of the family Lamiaceae, which are found in warm and mountainous areas. Among them, *O. vulgare* is one of the most widespread species in the Mediterranean region and western and southwestern Eurasia. *O. vulgare* is used in traditional medicine for various ailments, such as respiratory disorders, stomach aches, painful menstruation, rheumatoid arthritis, analgesia, nutritional disorders and urinary problems, being recognized as a diuretic and antiurolytic [16].

The Romanian-native *O. vulgare* ssp. *vulgare* extract was evaluated in terms of its phenolic composition by Oniga et al. [17], identifying gentisic, chlorogenic, p-coumaric and rosmarinic acids, hyperoside, isoquercitrin, quercitrin, quercetin and luteolin. At the same time, the extracts were proven to be strong cupric- and ferric-ion-reducing antioxidant agents, in good agreement with the total polyphenolic content. Parra et al. [18] evaluated the general composition of *O. vulgare* (adapted to northern Chile) extracts, revealing the presence of several bioactive compounds (rosmarinic acid, protocatechuic, gallic and ferulic acids, chlorogenic, luteolin, naringenin, carvacrol and thymol). The hydroalcoholic extract, obtained through an ultra-sound assisted method, revealed antioxidant and antimicrobial properties [18].

Rosmarinic acid is the main phenolic acid identified in the species *O. vulgare*. In this species, both free flavonoids (flavones, flavonols, flavanones and dihydroflavonols) and flavonoid glycosides are found. The concentration and distribution of compounds in *O. vulgare* can be influenced by the cultivar, environmental factors and different experimental protocols, making a detailed comparison between different reports difficult [19].

Different studies have evaluated the inhibitory effects of essential oil, extracts or main constituents of *O. vulgare* against different pathogenic bacteria. One of the mechanisms of antibacterial activity is the inhibition of the production or activity of bacterial enzymes (such as lipase and coagulase), mediated by the essential oils of *O. vulgare*. Another possible mechanism of action is the potential antibacterial synergy of the essential oil in combination with antibiotics to inhibit efflux pumps, which can be measured based on the fractional concentration index (FICI) [20].

In conclusion, hyssop and oregano represent two aromatic herbs that share several similarities. Both hyssop and oregano belong to the Lamiaceae family, with their flowers being similar in shape, forming whorls or spikes at the tops of the stems, displaying hues of pink, purple or white [21]. 

As also available for many other species from the Lamiaceae family, hyssop and oregano drew the attention of researchers working in the area of phytonanotechnology, particularly for in the phytosynthesis of silver nanoparticles [22,23,24]. 

The present work aims to comparatively evaluate the potential application of hyssop and oregano for the phytosynthesis of silver nanoparticles, as well as to evaluate the biological activities of their extracts and obtained nanoparticles, respectively, their antioxidant potential (in the DPPH assay) and their cytotoxicity, evaluated using human gingival fibroblasts (HFIB-G cell line).

## 2. Results

### 2.1. Extracts and NP Characterization

As a first step of the analytical protocol, the natural extracts were subjected to HPLC analysis, as well as to a determination of the total phenolic content. The results are presented in Table 1.

### 2.2. Characterization of Nanoparticles

**Figure 1:**
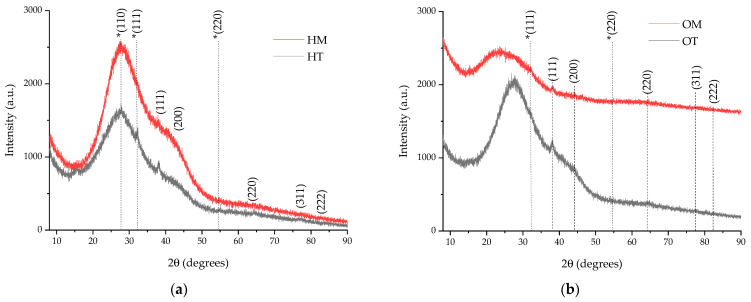
Diffractograms of the phytosynthesized nanoparticles: (**a**) NPs obtained using hyssop extracts (HM, HT); (**b**) NPs obtained using oregano extracts (OM, OT). The peaks marked with an asterisk (*) on the images represent diffraction planes attributed to the oxidation of the AgNPs to Ag_2_O (ICDD 00-012-0793) during sample preparation.

The phytosynthesis of the nanoparticles was confirmed using X-ray diffraction. The diffractograms of the analyzed samples are presented in Figure 1, while the data regarding the crystallite characteristics are presented in Table 2. In order to perform the analysis, the NP samples were centrifuged using a DLAB DM0408 laboratory centrifuge, at 4000 rpm, for two hours. The precipitate thus obtained was deposited on the glass support for analysis.

**Figure 2:**
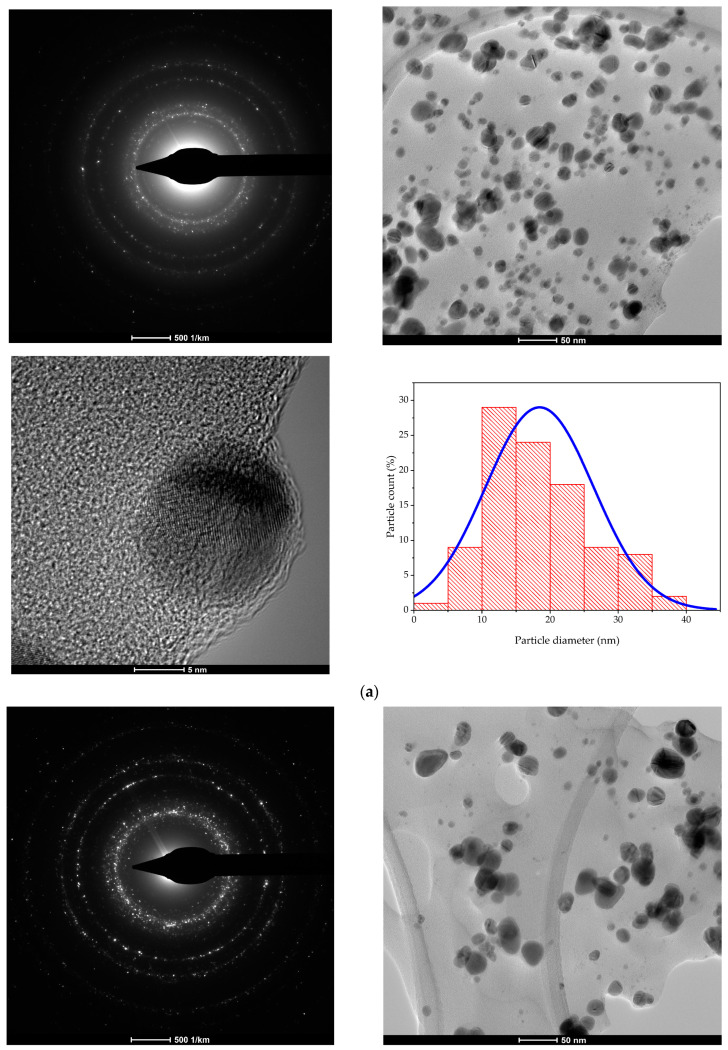

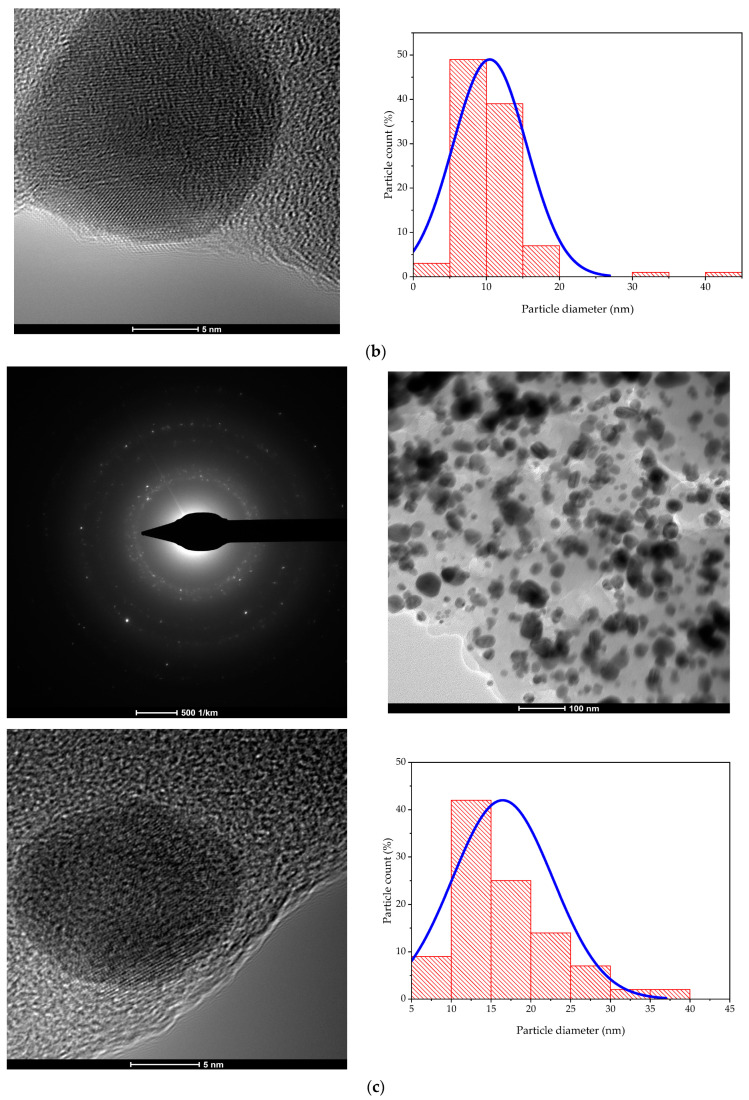

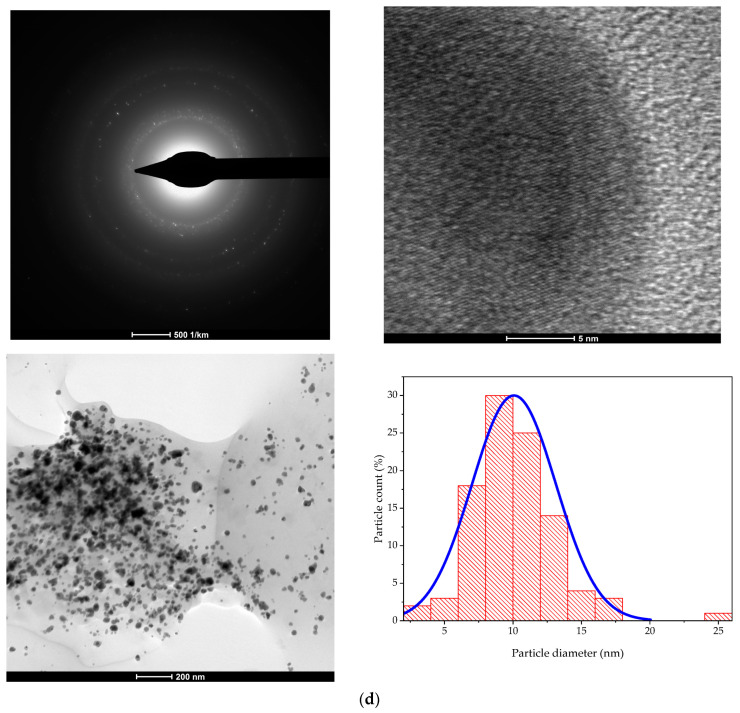
TEM images and size distribution obtained for the phytosynthesized nanoparticles: (**a**) sample HT; (**b**) sample HM; (**c**) sample OT; (**d**) sample OM.

For the evaluation of the NP morphologies, all the samples were subjected to TEM analyses. For analysis, a drop of extract containing the nanoparticle dispersion was diluted in a ratio of 1:10 (*v*/*v*), ultrasonated, subsequently placed in the centre of a copper grid, dried and subjected to microscopic analysis. The TEM results are presented in Figure 2. Nanoparticle size distribution was assessed via the direct measurements of nanoparticles from TEM images (over 150 measurements) using ImageJ image analysis software (v. 1.53u).

The TEM images revealed a quasi-spherical morphology for all the analyzed samples, while the distribution of their diameters (presented in Figure 2) was in good concordance with the XRD determinations: HT—approx. 18.5 nm, HM—10.5 nm, OT—16.5 nm, OM—10 nm.

### 2.3. Evaluation of Biomedical Potential and Cytotoxicity

**Figure 3:**
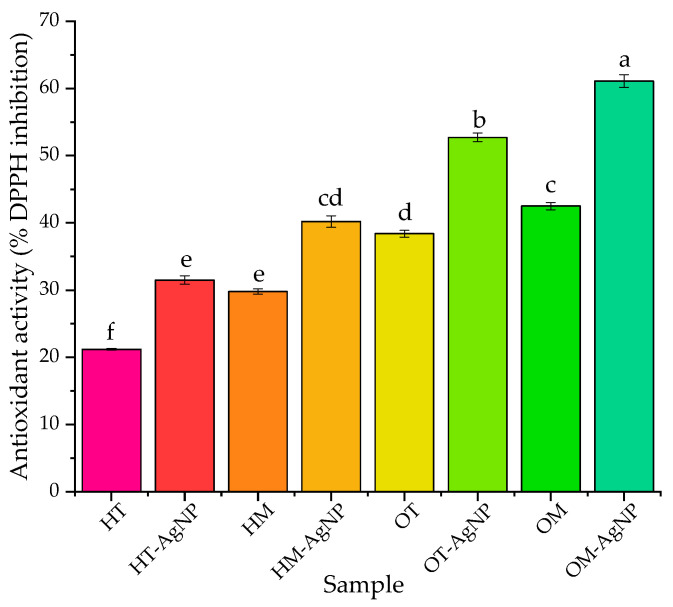
Results of the antioxidant DPPH inhibition assay. Values are means ± SEM, n = 5 per treatment group. Means without a common superscript letter differ (*p* < 0.05) as analyzed via one-way ANOVA and the TUKEY test.

The results of the antioxidant DPPH assay are presented in Figure 3. 

**Figure 4:**
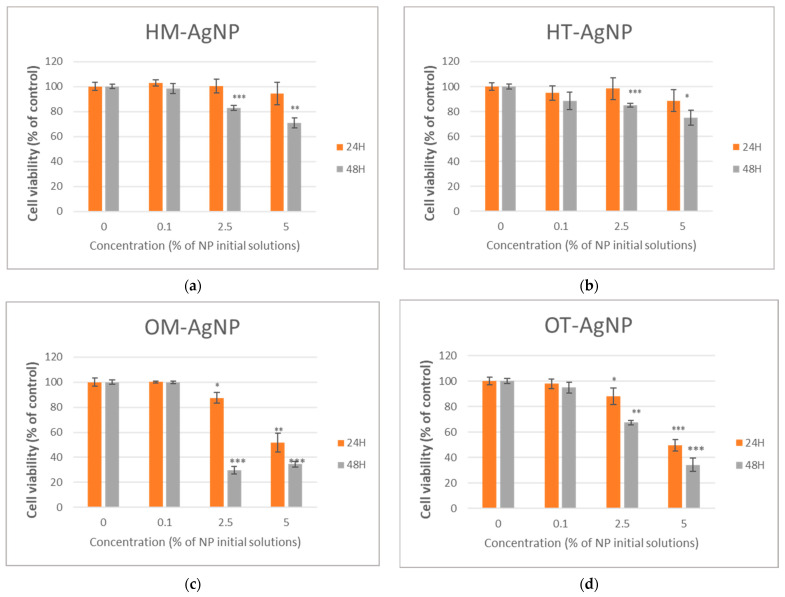
Cell viability measured by the MTT assay after the 24 and 48 h exposure of HFIB-G oral cells to different concentrations (expressed as % of initial NP solutions, according to the Materials and methods section) of HM-AgNP (**a**), HT-AgNP (**b**), OM-AgNP (**c**) and OT-AgNP (**d**). Values are the means ± SD (n = 3). * *p* < 0.05, ** *p* < 0.01 and *** *p* < 0.001 compared to control (untreated cells).

**Figure 5:**
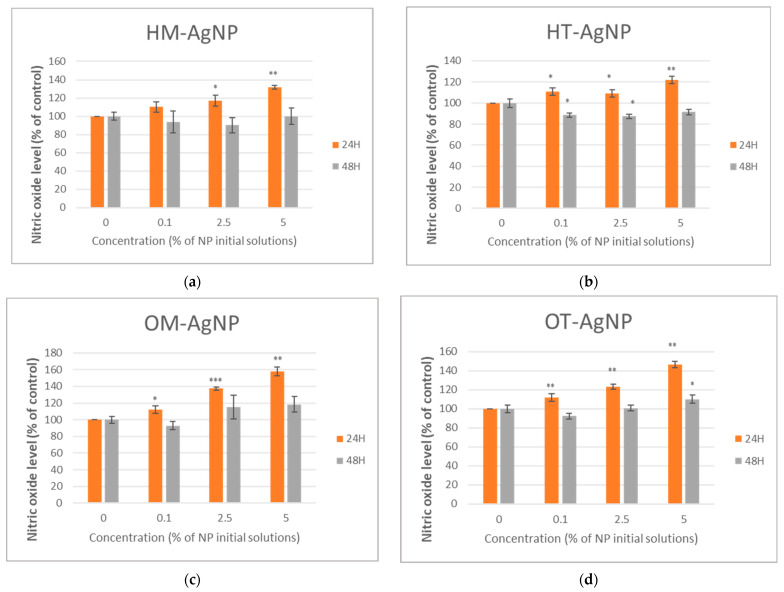
Nitric oxide level quantified after the 24 and 48 h incubation of HFIB-G oral cells with different concentrations (expressed as % of initial NP solutions, according to the Materials and methods section) of HM-AgNP (**a**), HT-AgNP (**b**), OM-AgNP (**c**) and OT-AgNP (**d**). Values are the means ± SD (n = 3); * *p* < 0.05, ** *p* < 0.01 and *** *p* < 0.001 compared to control (untreated cells).

**Figure 6:**
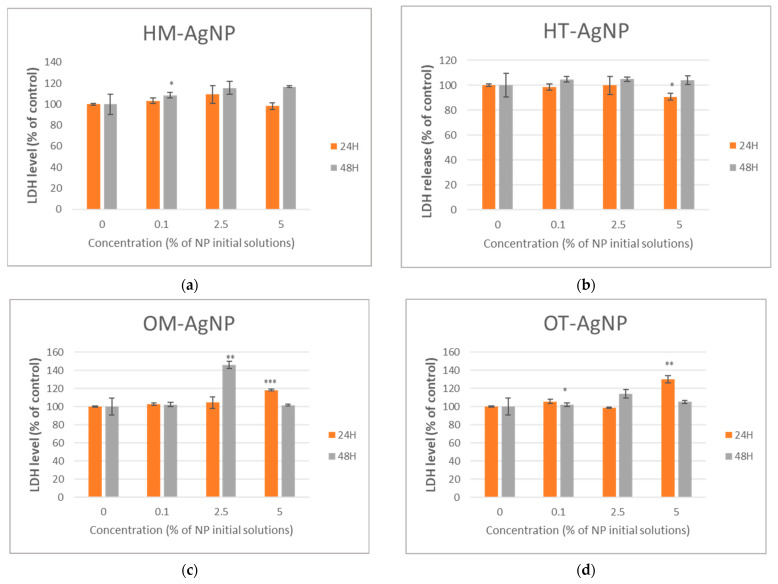
Level of lactate dehydrogenase released after the 24 and 48 h incubation of HFIB-G oral cells in the presence of different concentrations (expressed as % of initial NP solutions, according to the Materials and methods section) of HM-AgNP (**a**), HT-AgNP (**b**), OM-AgNP (**c**) and OT-AgNP (**d**). Values are the means ± SD (n = 3). * *p* < 0.05, ** *p* < 0.01 and *** *p* < 0.001 compared to control (untreated cells).

Cell viability, inflammation level and cytotoxicity are presented in Figure 4, Figure 5 and Figure 6.

After the MTT assay was performed, it was observed that tested phytosynthesized nanoparticles at the lowest concentration did not affect the level of cell viability after 24 and 48 h of incubation, but decreased it at higher concentrations, mostly after 48 h of incubation in comparison with the control (the cells without exposure to phytosynthesized nanoparticles). The highest concentration of HM-AgNP and HT-AgNP reduced the viability significantly, by 29.06%, respectively, and 25.2% after 48 h compared to the control (Figure 4a,b). In the same time, after 48 h of exposure to 5% (equivalent to approx. 27 μg Ag/mL) of OM-AgNP and OT-AgNP, the viability was drastically reduced by 65.45% and 65.79% compared to the control (Figure 4c,d), suggesting induced cytotoxicity in the tested cells.

Regarding the level of nitric oxide, an increasing tendency, in a dose-dependent manner after 24 h of incubation for both type of plants can be observed, compared to the control (Figure 5a–d), in accordance with the MTT results. In contrast, a decrease in the NO level was noticed after 48 h of exposure, compared to the control, especially for HM- AgNP (Figure 5a) and HT- AgNP (Figure 5b), suggesting the anti-inflammatory effects of the tested phytosynthesized nanoparticles exerted on the human gingival fibroblasts. The highest anti-inflammatory capacity was observed in the case of lower concentrations of HT-AgNP after 48 h of incubation, where the level of nitrite oxide decreased by approximately 12% compared to the control (Figure 5b), while in the case of AgNPs obtained using oregano extracts, a slight decrease in the NO level was noticed after 48 h of exposure, just at the lowest concentration, compared to the control (Figure 5c,d). It is also observed that the lowest nitrite level was recorded in the case of the samples obtained using the temperature extraction method (Figure 5a–d).

The LDH level was measured to observe the effect on cell membrane integrity after the exposure of HFIB-G cells to the tested phytosynthesized nanoparticles. Following exposure to HT-AgNP, the membrane integrity was not significatively affected (Figure 6b), while in case of incubation with HM-AgNP, it was, mostly after 48 h of incubation, with an approximate 15% increase in the LDH level compared to the control (Figure 6a). The highest increase was measured after 48 h of incubation with OM-AgNP (45.97% increase compared to control) (Figure 6c), while the highest level measured after incubation with OT-AgNP was 30.1% in comparison to the control, after 24 h (Figure 6d).

## 3. Discussion

The results of the phytochemical assays and HPLC analysis (Table 1) reveals the influence of the extraction method on the extract’s composition. Thus, the total phenolic compounds (expressed as mg GAE/g dried weight) are significantly higher for the microwave-assisted extraction; the oregano extracts have consistently higher TPC values, compared with the hyssop extracts. Literature data present some comparable results. For example, room temperature maceration in a hydroalcoholic mixture led to a TPC of approx. 2.45 mg GAE/g [25], while different extraction methods applied on oregano plant material led to TPC under 20 mg GAE/g [26]. 

Among the components identified via HPLC, for the group of phenolic acids (gallic, rosmarinic and protocatechuic acids), the sample HT presents significantly higher concentrations, compared with the other extracts. Also, the rosmarinic acid was identified in a higher content than other studies previously presented [27]. The flavanol group is constantly found in a higher concentration in the microwave extracts, as well as in the oregano extracts, with the same trend being recorded for the flavonoid standards (with the notable exception of naringenin), identified in higher quantities in the hyssop extracts, although, once again, the microwave-assisted extraction led to a superior yield. Significant exceptions were recorded for isoquercitrin (not detected in OT sample) and rutin (not detected in OM sample). An interesting case is represented by resveratrol. Identified in higher quantities in the hyssop extracts, it presents higher yields for the classical temperature extraction; this could be explained by its thermal lability, which can be accentuated in microwave-assisted techniques [28]. A similar discussion can also be made for ferulic acid (which can only be identified in the extracts obtained via temperature extraction). The other compounds recorded present variable trends, some being recorded only in some samples (i.e., protocatechuic acid was only recorded in the hyssop extracts, with hyperoside and luteolin in the oregano extracts). Rosmarinic acid, chlorogenic acid, caffeic acid, protocatechuic acid and isoquercitrin (all detected in the hyssop extracts, with the exception of rosmarinic acid, as previously discussed) were previously proven to vary in the hyssop hydroalcoholic extracts, depending on the environmental conditions (i.e., the administration of different concentrations of selenium) [29]. Similar observations were also previously published regarding the variation in different compounds also identified in the present study (rosmarinic acid, luteolin, caffeic acid, syringic acid, vanillic acid, gallic acid, naringenin), depending on *O. vulgare* accessions [30]. As such, all the results regarding the chemical composition of the extracts should be interpreted considering that, among the same plant species, significant compositional differences can occur, depending on the growing and harvesting parameters, as well as on the particular extraction method used [31]. In some cases, there can be observed an apparent inconsistency regarding the efficiency of the extraction method for particular compounds (caffeic acid, epicatechin, protocatechuic acid, rosmarinic acid), which are found in higher quantities in the hyssop extract obtained via classical temperature extraction (compared with the microwave extraction), while for the oregano extracts, the most efficient method was the microwave-assisted one. This can be explained by a selective extraction (using the applied parameters, and also considering the complex matrix of the plant materials) for some compounds, while for others, most probably, the applied extraction parameters were not fully optimized. The results are in line with other literature observations. For example, Dobrinčić et al. [32], comparing three extraction methods (microwave-assisted, ultrasound-assisted and high pressure-assisted extraction) for the separation of polyphenols from olive leaves, observed significant differences for several target compounds: oleuropein was obtained in the highest quantity in the microwave-assisted extraction—MAE, while caffeic acid was obtained in the highest quantity using the ultrasound-assisted method—UAE (for the same parameters used—MAE 2 min, 80 °C, 3 g of plant material/UAE 7 min, 100% amplitude, 3 g of plant material). When varying the parameters of the extraction methods (MAE 8.5 min, 45 °C, 1.5 g of plant material/UAE 14 min, 50% amplitude, 1.5 g of plant material), the oleuropein was found in higher quantities in the extract obtained using the ultrasound-assisted method, while the caffeic acid was found in higher quantities in the extract obtained via MAE [32]. 

X-ray diffraction data support the phytosynthesis of silver nanoparticles in the cubic crystalline system, showing the four diffraction maxima corresponding to the diffraction planes (111)—approx. 38°, (200)—approx. 44°, (220)—approx. 64°, respectively (311)—approx. 77°. The identification was made based on ICDD entry 01-087-0719. Some secondary diffraction planes, corresponding to Ag_2_O (ICDD 00-012-0793), are most probable due to the oxidation of the samples during sample preparation for analysis.

The XRD data (Table 2) can be correlated, to some extent, with the total phenolic content (Table 1); thus, the microwave-assisted extracts consistently led to smaller-crystallite-dimension nanoparticles, a trend that was also previously observed for *L. cardiaca* extracts [31]. The crystallite size, as determined by the Scherrer equation, although it does not represent a true particle dimension determination (considering all the various factors influencing the determination, among which it can be mentioned the strain, equipment errors, residual stress, etc.), can provide valuable information on the NP characteristics. 

The most valuable method for the evaluation of both morphology and dimensions, when we are speaking of nanoparticles, is represented by the transmission electron microscopy. The TEM analyses (Figure 2) support the claim regarding the phytosynthesis of quasi-spherical nanoparticles (some other morphologies, such as triangular or ellipsoidal, being also encountered) and confirm the synthesis of nanoparticles with spherical or quasi-spherical morphologies (although other types of morphologies are also encountered, such as triangular, hexagonal or ellipsoidal). The size distribution of the nanoparticles (as determined by direct measurements from over 100 NPs) suggests average diameters between 10 and 20 nm, with the lowest dimensions being recorded for microwave-assisted oregano extract NPs (OM). Also, the dimensions recorded in the case of hyssop extract NPs present the same trend, with smaller dimensions recorded for HM.

Considering the differences recorded in TPC values and HPLC results (Table 1), it can be concluded that other groups of secondary metabolites also contribute to the phytosynthesis process, thus leading to the observed results.

The results regarding the phytosynthesized NPs are in line with relevant literature data. For example, Sankar et al. [22], using an aqueous oregano leaf extract obtained nanoparticles with a crystallite size of approx. 65 nm (determined via XRD) and 63–85 nm (via field emission-scanning electron microscopy). Shaik et al. [23], using a similar aqueous aerial part extract, obtained average diameters of approx. 12 nm (via transmission electron microscopy), with no crystallite size being reported. Balciunaitiene et al. [24] compared the potential of hyssop leaf hydroalcoholic obtained via maceration with a similar calendula extract. The authors observed a superior potential towards the phytosynthesis of silver nanoparticles for the hyssop extract (average diameter 16.8 nm) compared with the calendula extract (average diameter 35.7 nm), as determined via TEM.

Evaluating the results of the DPPH antioxidant assay, an increase in antioxidant activity following the same trend as the TPC variation can be noticed. Also, the phytosynthesis process leads to the formation of NP dispersions with significantly higher antioxidant activity, compared with the corresponding extracts used as precursors. As another general conclusion, the oregano extracts possess significantly higher antioxidant potential, as well as microwave-assisted extracts (compared with classical temperature extracts), in the DPPH assay.

Many studies highlighted that the natural plant extracts revealed a dual behavior, firstly acting as capping and reducing agents, and, furthermore, modulating the NPs’ biological effects in a similar way [33,34,35]. Medicinal plants, such as *Senna siamea*, *Medicago sativa*, *Aloe vera*, *Azadirachta indica*, *Mentha piperita*, *Ananas comosus* and many others, have already been used for the development of AuNPs and AgNPs with biomedical potential [36,37,38,39,40,41,42]. However, the plant kingdom still offers a great number of potential alternatives for the phytosynthesis process. In our study, we aimed to investigate the biocompatibility of hyssop and oregano photosynthesized silver nanoparticles, in human gingival fibroblasts, in order to evaluate their potential biomedical applications, with no similar studies being performed to date, to our knowledge, regarding the biocompatibility of hyssop and oregano photosynthesized silver NPs in these types of cells. 

Our results revealed no significant changes in viability (MTT assay) levels for both types of AgNPs, HM-AgNPs and HT-AgNPs, at the lowest concentration, compared to the *control* (Figure 4a,b). However, the incubation of human gingival fibroblasts for 48 h with concentrations of 2.5% and 5% (of initial NPs solutions), for both types of H-AgNPs, triggered a significant decrease in cell viability compared to the control (Figure 4a,b). Moreover, in the case of the cells incubated for 48 h with HM-AgNPs, the LDH level was significantly increased when compared with the control, being in accordance with the MTT results (Figure 6a,b). Our results showed that 24 h of incubation with 2.5% and 5% HM-AgNPs induced significant increases in NO levels. Furthermore, such increases were also observed after 24 h of incubation with HT-AgNPs, at all three studied concentrations. Comparing the effects after 24 h of incubation with the two types of NPs, our data revealed a significant increase in the NO level in the case of HM-AgNPs *versus* HT-AgNPs. These data concerning the NO levels suggest possible proinflammatory effects triggered by the cells’ exposure to the two types of NPs in the first 24 h. A preliminary conclusion of our study suggests the good biocompatibility of HT-AgNPs, especially for the 0.1% concentration and 24 h incubation time. These findings might open the way towards the applicability of HT-AgNPs in developing nanomaterial-based solutions for inflammatory oral diseases.

Data from our study interestingly revealed that the incubation of human gingival fibroblasts for 24 h with both types of O-AgNPs, at concentrations of 2.5% and 5%, induced significant decreases in cell viability (MTT assay), directly proportional with NPs concentrations (Figure 4c,d). The most notable viability decrease was triggered by 5% O-AgNPs. Similar results were obtained for the 48 h incubation time. According to the MTT results, our data have revealed that after 24 h of incubation of the cells with 5% OM-AgNPs and 5% OT-AgNPs, respectively, a significant increase in LDH levels was induced, compared with the control, suggesting a cytotoxic effect of both types of NPs at this concentration. Considering the results for LDH obtained after a 48 h incubation, we might outline the idea that after the first 24 h, an adaption mechanism is initiated in the presence of both types of O-AgNPs. Moreover, a 24 h incubation of the human gingival fibroblasts with OM-AgNPs at all three tested concentrations induced significant increases in NO levels, in a concentration-dependent manner. Similar results were obtained for OT-AgNPs. The most notable NO level increment was obtained after 24 h of incubation with 5% OM-AgNPs. These results might suggest the possible pro-inflammatory effects of cell exposure to both types of NPs at all three tested concentrations. Our data also illustrates a possible adaptation mechanism, for which the effects become visible after a 48 h incubation. Taking all of these data into account, we can outline the conclusion that in the case of *O. vulgare*, the best biocompatibility was obtained for 0.1% OT-AgNPs for a 48 h incubation. Similar *O. vulgare* biocompatibility results were reported by Benedec D. et al., with the difference that their study was conducted on dermal fibroblasts exposed to oregano AuNPs [43].

Comparing the biocompatibility illustrated based on our results for the two plant species, we can outline the conclusion that phytosynthesized *H. officinalis* as 0.1% HT-AgNPs might be more suitable for oral inflammatory disease treatment than AgNPs obtained using *O. vulgare* extracts.

## 4. Materials and Methods

### 4.1. Materials

The vegetal material used for the studies are represented by certified hyssop and oregano aerial parts, commercially available, and purchased from a local market. 

In order to obtain natural extracts, two extraction methods were applied (classical temperature extraction and microwave-assisted extraction). The extraction parameters and extract encodings are presented in Table 3. For all the extraction procedures, the ratio of vegetal material/solvent was kept at 1/10 (*w*/*v*). All the extracts obtained were filtered using filter paper The phytosynthesis procedure involved the mixing of equal quantities of filtered natural extracts and a silver nitrate solution (10^−3^ M, Chimreactiv, Bucharest, Romania). The ethanol used for the extraction procedures was reagent quality (Chimreactiv, Bucharest, Romania), while the bidistilled water used for all experiments was obtained in the laboratory, using a GFL 2102 water still (GFL, Burgwedel, Germany).

### 4.2. Characterization Methods

The composition of the natural extracts was evaluated using one phytochemical assay (determination of the total content of phenolic compounds) and high-precision liquid chromatography (HPLC, for the quantification of several target compounds). All reagents were used as received, without further purification.

The total phenolics content was determined using a colorimetric method (Folin–Ciocâlteu reagent method), previously described [31], involving the reduction of the Folin–Ciocâlteu reagent by phenolic compounds, with the formation of a blue complex. The Folin–Ciocâlteu reagent (Merck KGaA, Darmstadt, Germany) and sodium carbonate solution (Merck KGaA, Darmstadt, Germany) were commercially available and used without any purification; the optical density was determined at 765 nm using an Rigol Ultra 3660 UV-Vis spectrophotometer (Rigol Technologies, Beijing, China), with the determined values being compared to a standard curve prepared with gallic acid solutions, and the final results being expressed as milligrams of gallic acid equivalents (GAE)/gram of dried matter [31]. 

Five determinations were performed, with the results being presented as the average of the determinations ± the standard error of the mean.

The quantification of polyphenols and the other compounds present in the extracts was carried out using an L-3000 HPLC system (Rigol Technologies Inc., Beijing, China) equipped with a diode-array detector (HPLC-DAD) and a Kinetex EVO C18 column (150 × 4.6 mm, particle size of 5 µm). The mobile phase consisted of a system with two solvents, and the elution was carried out in gradient mode. The solvents used were (A) 0.1% trifluoroacetic acid (TFA) in water and (B) 0.1% TFA in acetonitrile. The elution gradient was as follows: 2–100% B at 30 °C for 60 min at an elution flow rate of 1 mL/min. The analysis was performed at 5 different wavelengths (255, 280, 325 and 355 nm) in accordance with the specialized literature. The stock solutions containing the reference compounds (belonging to several classes: phenolic acids—gallic acid, protocatechuic acid, rosmarinic acid, flavanols—catechin, epicatechin, hyperoside, flavonoids—isoquercitrin, rutin, naringin, luteolin, naringenin, benzoic acid derivatives—vanillic acid, hydroxycinnamic acids and derivatives—caffeic acid, ferulic acid, sinapic acid, p-coumaric acid, o-coumaric acid, tannins and derivatives—tannic acid, ellagic acid, chlorogenic acids—chlorogenic acid, gallic acids and derivatives—syringic acid, phytoalexins—resveratrol, all standards Merck KGaA, Darmstadt, Germany) were prepared so that their concentration was 1000 µg/mL. For the calibration curves, concentrations between 10 and 400 µg/mL were used.

X-ray diffraction (XRD) analyses were performed using a 9 kW Rigaku SmartLab diffractometer (Rigaku Corp., Tokyo, Japan, operated at 45 kV and 200 mA, CuKα radiation—1.54059 Å), in scanning mode 2θ/θ, between 7 and 90° (2θ). Components were identified based on a comparison with ICDD data.

The crystallite size was determined using the Debye–Scherrer equation:

(1) $$Dp=\frac{\left(K\times \lambda \right)}{\left(\beta \times cos\theta \right)}$$

where *Dp* = the average size of the crystallites, *K*—the Scherrer constant (for cubic structures, *K* = 0.94), β = represents the width at half-height of the diffraction maximum, *θ* = the Bragg angle, *λ* = the wavelength—1.54059Å, in our case.

Transmission Electron Microscopy images were recorded using a Titan Themis 200 image corrected transmission electron microscope (FEI, Hillsboro, OR, USA), equipped with a high-brightness field emission gun (X-FEG) electron source and a Super-X detector for energy dispersive spectroscopy (EDX). The heterostructures were investigated at 200 kV via HR-TEM, coupled with selected area electron diffraction (SAED) and scanning transmission electron microscopy (STEM) for elemental line profiling.

### 4.3. Evaluation of Biomedical Potential and Cytotoxicity

The antioxidant activity of the extracts and silver nanoparticles was determined using the DPPH assay (Sigma Aldrich, Burlington, MA, USA) [31]. The antioxidant activity (*AA%*) was calculated using the following formula:

(2) $$AA\%=\frac{\left(A_{blank}-A_{sample}\right)}{A_{blank}}\times 100$$

where *A_blank_* = absorbance of DPPH solution without sample, A_sample_ = absorbance of extract mixed with 0.02 mg/mL DPPH solution.

Five determinations were performed, with the results being presented as the average of the determinations ± the standard error of the mean.

Cell Culture: Human fibroblasts, gingiva (HFIB-G cell line, cat. -no.: 1210412, from Provitro, Berlin, Germany) were grown in complete Dulbecco’s Modified Eagle’s Medium (DMEM) supplemented with 10% fetal bovine serum (FBS) and 1% penicillin/streptomycin/amphotericin. The cells were maintained in a humidified atmosphere with 5% CO_2_, at 37 °C. The cells (10^4^ cells/well) were seeded in 96-well plates and left to adhere overnight. Then, the fibroblasts were exposed to HT-AgNP, HM-AgNP, OT-AgNP or OM-AgNP for the next 24 and 48 h at 37 °C with 5% CO_2_. The samples were previously diluted in complete Dulbecco’s modified Eagle’s medium, and the obtained concentrations of the phytosynthesized NPs were 0.1% (equivalent with a concentration of approx. 0.54 µg Ag/mL), 2.5% (equivalent with a concentration of 13.5 µg Ag/mL) and 5% (equivalent with a concentration of approx. 27 µg Ag/mL). The final volume was 300 µL for each well. Control samples were represented by cells unexposed to phytosynthesized NPs. At the end of each incubation time, the cells were examined on an inverted microscope Optika IM-3. 

Cell Viability Assay: viability of the cells was determined using the 3-(4,5-dimethylthiazol-2-yl)-2,5-diphenyltetrazolium bromide (MTT, Thermo Fisher Scientific, Eugene, OR, USA) assay which is based on the reduction of the yellow tetrazolate salt (MTT) to violet-colored formazan crystals in the viable cells (Hansen; Nielsen; Berg, 1989, Re-examination and further development of a precise and rapid dye method for measuring cell growth/cell kill). The growth medium was removed after 24 and 48 h of incubation, and then, 100 µL of DMEM with 10 µL MTT was added to each well for 4 h at 37 °C. After incubation, 100 µL/well of SDS–HCl solution (10%) was used to dissolve the purple formazan crystals, and after another 4 h, absorbance was measured at 570 nm with a FLUOstar^®^ Omega multi-mode microplate reader from BMG LABTECH (Ortenberg, Germany).

Griess Assay: the NO levels accumulated in the cell culture medium were measured after 24 and 48 h of incubation using the Nitric Oxide Assay Kit (NO, Thermo Fisher Scientific, Vienna, Austria) based on Griess reagent (0.1% naphthyl ethylenediamine dihydrochloride and 1% sulphanilamide in 5% H_3_PO_4_). The absorbance at 540 nm was measured using a BMG LABTECH’ FLUOstar^®^ Omega multi-mode microplate reader (Ortenberg, Germany), and the results were compared to the control. Increased NO levels are produced from reduced nitrites during inflammation and related to the cytotoxic effects.

Lactate Dehydrogenase (LDH) Assay: LDH (Thermo Fisher Scientific, Eugene, OR, USA) was used to measure released LDH from the damaged cells after 24 and 48 h of cell growth in the presence of phytosynthesized NPs. To perform the assay, 50 µL of each sample medium was mixed with 50 µL of reaction mixture (substrate and cofactor) and incubated at room temperature for 30 min in the dark. To stop the reaction, 50 µL of stop solution was added, and the absorbance was measured at 490 nm and 680 nm using a FLUOstar^®^ Omega multi-mode microplate reader from BMG LABTECH (Ortenberg, Germany). To determine LDH activity, the 680 nm absorbance was subtracted from the 490 nm absorbance, and the results were compared to the control. 

### 4.4. Statistical Interpretation and Data Representation

The determinations carried out based on multiple parallel determinations (as mentioned for each method) and the data obtained were analyzed for statistical significance using an analysis of variance (one-way ANOVA) and Tukey’s test to determine significant differences between means. Significant differences were set at *p* ≤ 0.05. Results shown are the mean ± standard error of the mean (SE) of independent determinations. The results of the MTT, NO and LDH assays were expressed as averages with standard deviations. Quantitative independent variables were tested between groups using Student *t*-Tests. The *p* < 0.05 values were considered statistically significant.

Graphical representations were constructed using the OriginPro 2018 Data Analysis and Graphing Software (OriginLab Corporation, Northampton, MA, USA).

## 5. Conclusions

The results of the study support the use of hyssop and oregano extracts for the phytosynthesis of silver nanoparticles. Using two different extraction methods (classical temperature extraction and microwave-assisted extraction), natural extracts were obtained with significant compositional differences. More than that, these differences, in terms of composition, influence the morphologies and dimensions of the developed nanoparticles, with the microwave-assisted method leading to smaller dimensions, while the oregano extracts led to smaller dimensions, compared with a similar method for hyssop extracts (both in crystallite size determined via XRD and average in diameter, determined via TEM). 

These characteristics (extract composition and nanoparticle dimensions) also influence the antioxidant potential, with the best DPPH inhibition being recorded for the oregano microwave-assisted extract-NPs (over 60% inhibition). 

The obtained results revealed no significant changes in viability (MTT assay) levels for both types of AgNPs, HM-AgNPs and HT-AgNPs, at the lowest concentration, compared to the control, while the incubation of human gingival fibroblasts for 48 h with higher concentrations triggered a significant decrease in cell viability. Moreover, in the case of the cells incubated for 48 h with HM-AgNPs, the LDH level was significantly increased when compared with the control, being in accordance with the MTT results. After 24 h of incubation with the higher concentration HM-AgNPs, a significant increase in NO levels was observed, with increases also observed after 24 h of incubation with HT-AgNPs, at all studied concentrations. Comparing the effects after 24 h of incubation with the two types of NPs, our data revealed a significant increase in the NO level in the case of HM-AgNPs *versus* HT-AgNPs. These data concerning the NO levels suggest possible proinflammatory effects triggered by the cells’ exposure to the two types of NPs in the first 24 h. A preliminary conclusion of our study suggests the good biocompatibility of HT-AgNPs, especially for the 0.1% concentration and 24 h incubation time. The results obtained revealed that the incubation of human gingival fibroblasts for 24 h with both types of oregano AgNPs, at higher concentrations, induced significant decreases in cell viability (MTT assay), directly proportional with NP concentrations, while the results for LDH obtained after a 48 h incubation outline the idea that after the first 24 h, an adaption mechanism is initiated in the presence of both types of oregano AgNPs. Moreover, a 24 h incubation of the human gingival fibroblasts with OM-AgNPs at all three tested concentrations induced significant increases in NO levels, in a concentration-dependent manner. The best biocompatibility was obtained for oregano-NPs for a 0.1% concentration of OT-AgNPs during a 48 h incubation.

## Figures and Tables

**Table 1:** Evaluation of extract composition via HPLC and total phenolic content ^1^.

Extract/Parameter	HT	HM	OT	OM
TPC (mg GAE/g dried matter)	10.9 ± 0.0224 ^d^	14.4 ± 0.0358 ^c^	23.4 ± 0.0626 ^b^	28.4 ± 0.0716 ^a^
Tannic acid (mg/L)	N.D.	9 ± 0.00635 ^a^	1.07 ± 0.000577 ^b^	0.902 ± 0.000577 ^c^
Gallic acid (mg/L)	0.166 ± 5.77 × 10^−5 c^	0.239 ± 0.000173 ^a^	0.186 ± 0.000115 ^b^	0.154 ± 5.77 × 10^−5 d^
Protocatechuic acid (mg/L)	4.88 ± 0.00346 ^a^	0.512 ± 0.000346 ^b^	N.D.	N.D.
Catechin (mg/L)	N.D.	N.D.	1.62 ± 0.00115 ^b^	10.4 ± 0.00693 ^a^
Vanillic acid (mg/L)	0.0709 ± 0.000115 ^b^	N.D.	N.D.	1.45 ± 0.000577 ^a^
Caffeic acid (mg/L)	28.9 ± 0.0173 ^a^	22.2 ± 0.0144 ^c^	15.2 ± 0.00577 ^d^	23.1 ± 0.0162 ^b^
Ellagic acid (mg/L)	5.44 ± 0.00346 ^b^	6.45 ± 0.00404 ^a^	3.2 ± 0.00173 ^c^	0.182 ± 0.000115 ^d^
Chlorogenic acid (mg/L)	2.76 ± 0.00173 ^c^	3.12 ± 0.00173 ^b^	0.15 ± 0.000115 ^d^	9.7 ± 0.00635 ^a^
Syringic acid (mg/L)	3.48 ± 0.00231 ^d^	4.03 ± 0.00289 ^c^	5.34 ± 0.00346 ^a^	4.94 ± 0.00346 ^b^
Epicatechin (mg/L)	5.62 ± 0.00404 ^c^	2.38 ± 0.00173 ^d^	21.8 ± 0.0139 ^b^	59.3 ± 0.0433 ^a^
p-coumaric acid (mg/L)	2.47 ± 0.00173 ^b^	N.D.	N.D.	4.77 ± 0.00289 ^a^
Ferulic acid (mg/L)	7.7 ± 0.0052 ^a^	N.D.	7.33 ± 0.0052 ^b^	N.D.
Sinapic acid (mg/L)	13.1 ± 0.0924 ^c^	13.8 ± 0.00866 ^b^	N.D.	29.5 ± 0.0202 ^a^
o-coumaric acid (mg/L)	1.52 ± 0.00115 ^d^	1.74 ± 0.00115 ^c^	2.38 ± 0.00115 ^a^	1.91 ± 0.00115 ^b^
Isoquercitrin (mg/L)	7.42 ± 0.00462 ^c^	12.2 ± 0.00866 ^b^	N.D.	95.2 ± 0.0658 ^a^
Rutin (mg/L)	94.4 ± 0.0635 ^c^	124 ± 0.086 ^b^	273 ± 0.191 ^a^	N.D.
Hyperoside (mg/L)	N.D.	N.D.	65.4 ± 0.0462 ^b^	243 ± 0.168 ^a^
Naringin (mg/L)	12.9 ± 0.00866 ^d^	16.7 ± 0.0115 ^c^	20.2 ± 0.0115 ^b^	28.7 ± 0.0289 ^a^
Rosmarinic acid (mg/L)	4380 ± 3.04 ^a^	N.D.	434 ± 0.3 ^c^	765 ± 0.538 ^b^
Resveratrol (mg/L)	83.8 ± 0.0577 ^a^	5.9 ± 0.00404 ^c^	47.5 ± 0.0346 ^b^	N.D.
Luteolin (mg/L)	N.D.	N.D.	49.8 ± 0.0341 ^b^	55.3 ± 0.0416 ^a^
Naringenin (mg/L)	4.19 ± 0.00289 ^b^	6.07 ± 0.00404 ^a^	1.3 ± 0.000924 ^d^	1.82 ± 0.00173 ^c^

^1^ Values represent the mean of five determinations (for TPC), respectively, three determinations (for HPLC results) ± SE; values in the same row without a common superscript letter differ statistically (*p* < 0.05) as analyzed via one-way ANOVA and the TUKEY test; N.D.—not detected: sample encoding: HT—hyssop extract obtained via classic temperature extraction; HM—hyssop extract obtained via microwave-assisted extraction; OT—oregano extract obtained via classic temperature extraction; OM—oregano extract obtained via microwave-assisted extraction.

**Table 2:** The position of the diffraction maxima and the crystallite size for the analyzed samples, calculated according to Equation (1).

Sample	(111) Peak Position (Degrees)	(200) Peak Position (Degrees)	(220) Peak Position (Degrees)	(311) Peak Position (Degrees)	FWHM (Degrees) ^1^	Crystallite Dimension (nm) ^1^
HT	38.0988	43.24	64.70	77.42	1.2738	6.89
HM	38.0264	43.15	64.41	77.54	2.7291	3.21
OT	38.0988	44.41	64.23	77.32	2.149	4.08
OM	38.0531	44.25	64.04	77.88	2.9862	2.94

^1^ Data for (111) diffraction plane; sample encoding: HT—hyssop extract obtained via classic temperature extraction; HM—hyssop extract obtained via microwave-assisted extraction; OT—oregano extract obtained via classic temperature extraction; HM—hyssop extract obtained via microwave-assisted extraction; OM—oregano extract obtained via microwave-assisted extraction.

**Table 3:** Extraction procedures followed and encodings of the obtained materials.

Vegetal Material	Extraction Method	Extraction Parameters	Extract Encoding	Phytosynthesized NPs
*Hyssopus officinalis* L., aerial parts	Temperature extraction	Shredded vegetal material, extracted for 3 h at 65 °C, solvent—hydroalcoholic mixture (ratio of ethanol:water = 1:1)	HT	HT-AgNP
	Microwave-assisted extraction	Crushed vegetal material and hydroalcoholic solvent (ratio of ethanol:water = 1:1) heated using an Ethos Easy Advanced Microwave Digestion System (Milestone Srl, Sorisole, Italy), extraction time 25 min, extraction temperature 65 °C, microwave power 800 W	HM	HM-AgNP
*Origanum vulgare* L., aerial parts	Temperature extraction	Shredded vegetal material, extracted for 3 h at 65 °C, solvent—hydroalcoholic mixture (ratio of ethanol:water = 1:1)	OT	OT-AgNP
	Microwave-assisted extraction	Crushed vegetal material and hydroalcoholic solvent (ratio of ethanol:water = 1:1) heated using an Ethos Easy Advanced Microwave Digestion System (Milestone Srl, Sorisole, Italy), extraction time 25 min, extraction temperature 65 °C, microwave power 800 W	OM	OM-AgNP

## Data Availability

The data presented in this study are available on request from the corresponding authors.
